# Effects of sex, toilet training, stress, and caffeine on nocturnal enuresis among school children in Gondar Town, the metropolitan city of Ethiopia: a community-based study in 2023

**DOI:** 10.3389/fped.2024.1366430

**Published:** 2024-06-10

**Authors:** Nega Tezera Assimamaw, Atnasiya Kibkab Kebede, Kalkidan Bazezew Genetu

**Affiliations:** ^1^ Department of Pediatrics and Child Health Nursing, College of Medicine and Health Sciences, University of Gondar, Gondar, Ethiopia; ^2^ University of Gondar Comprehensive Specialized Hospital Gondar, Gondar, Ethiopia; ^3^ Department of Surgical Nursing, College of Medicine and Health Sciences, University of Gondar, Gondar, Ethiopia

**Keywords:** children, Gondar, Ethiopia, nocturnal enuresis, predictors

## Abstract

**Background:**

Nocturnal enuresis is associated with severe social and psychological problems that affect one's self-esteem, later in life, harmed adolescent and adult life, emotional stress on the family, and poor school performance. Moreover, enuresis children may cause panic attacks, mood disorders, and depression. This study aims to assess the prevalence and associated factors of nocturnal enuresis among children aged 5–14 years in Gondar city, Northwest Ethiopia, 2023.

**Methods:**

A community-based, cross-sectional study was conducted from April 1, 2023, to May 30, 2023. A stratified multistage sampling technique was used to select study subject from kebeles in Gondar city. The data were collected by using a structured, interviewer-administer Questionnaire. The data were entered using EPI DATA version 4.6.02 software, and processed,and analyzed using the statistical package for the social sciences (SPSS) version 25. All variables with *P* ≤ 0.25 in the bivariate analysis were included in the final model of multivariate analysis. The multivariate binary logistic regression was used to assess the association between the independent and outcome variable. The direction and strength of statistical association were measured with an adjusted odds ratio along with 95% CI and a *P*-value <0.05 was considered statistically significant.

**Result:**

The overall prevalence of nocturnal enuresis among children aged 5–14 years was 162 (22.2%). The findings showed that being boys [AOR = 0.54; 95% CI (0.31, 0.93)], child and no toilet training practices [AOR = 2.50; 95% CI (1.02, 6.15)], Having no caffeine [AOR = 0.16; 95% CI (0.09, 0.29)], and exposure to stressful events [AOR = 20; 95% CI (11.12, 33.34)] had a significant association with nocturnal enuresis, *p*-value <0.05.

**Conclusion:**

In this study, the prevalence of nocturnal enuresis children age 5–14 years was higher than that in previous studies. Sex of child, toilet training practices, caffeine c before bed, and presences of stressful event were a significant predictor of nocturnal enuresis.

## Introduction

According to the International Children’s Continence Society (ICCS) defined as nocturnal enuresis (NE) is characterized by the intermittent involuntary release of urine during sleep, regardless of whether the child experiences daytime urinary symptoms (1).

NE is the medical term used to describe the condition of involuntary urination during sleep in children who are at least five years old. It is commonly referred to as bedwetting. Nocturnal enuresis can occur in two forms such as primary and secondary. Primary nocturnal enuresis refers to bedwetting that has been ongoing since childhood without a significant period of dryness, whereas secondary nocturnal enuresis occurs when a child who has previously achieved nighttime dryness begins to wet the bed again (2). It is a common childhood problem. In the community as well as by clinicians, bed-wetting is frequently viewed as a psychological problem (1, 3).

The prevalence of nocturnal enuresis can vary across the different studies conducted in different regions of the world. Different studies have reported varying prevalence rates ranging from as low as 3.9% to as high as 17% in China and Turkey respectively (4, 5).

According to studies conducted in the African continent, particularly in South Africa and Egypt, the prevalence rates are relatively high, at 16.0% (6) and 18% (7) respectively. Moreover, the studies conducted in different parts of Ethiopia have shown relatively high prevalence rates of 20.8% (8) and 26.6% (9), r. These findings suggest that NE is a significant concern in Ethiopia, and the prevalence rates reported in these studies are higher compared to some other regions.

NE can be a frustrating clinical issue for both children and their parents. It can have a significant impact on the emotional well-being and quality of life of those affected. It is considered one of the most prevalent clinical issues in children, particularly during the early years of childhood (10). The illness ultimately has an effect on the kids’ social lives and results in secondary emotional and academic issues (10).

Nocturnal enuresis (NE) can lead to various emotional and psychosocial complications like feelings of guilt and shame in individuals, particularly children. They may blame themselves for their inability to control their bladder during sleep, which can negatively impact their self-esteem, may feel isolated or different from their peers. They may be reluctant to participate in overnight activities, such as sleepovers or camps, fearing embarrassment or ridicule from their peers. This can result in social withdrawal or limited social interactions, anxiety, and depression. The stress and frustration associated with recurring bed-wetting episodes can take a toll on an individual's emotional well-being and significantly impact self-esteem. Children may feel inadequate or inferior to their peers who do not experience bed-wetting. This can affect their confidence and overall self-perception, may become targets of teasing, bullying, or victimization by their peers. This can further exacerbate feelings of shame and social isolation (11–14).

Nocturnal enuresis, or bed-wetting, can be a significant developmental issue for school-age children, and it can have various impacts on both the child and the family (15, 16). Some of the effects include it can also cause emotional stress within the family. Parents may feel concerned, frustrated, or even blame themselves for their child's bed-wetting (17). Siblings may also be affected, as attention and resources may be directed toward managing the bed-wetting issue. The ongoing management of bed-wetting can create additional stress within family dynamics.

In addition to the stress of caring for an enuresis child, parents of children with the condition had additional work to do and laundry expenses to pay (18). According to studies, 30% of parents with enuresis experience their children developing enuresis intolerance (18). Furthermore, nocturnal enuresis had severe social and psychological repercussions that affected one's self-worth, academic achievement, parental approval, and later in life, one's ability to have a sexual problem (19). It has been discovered that children who are diagnosed with enuresis lack self-confidence compared to those who have no nocturnal enuresis, and when they are ill with some serious diseases, the results of nocturnal enuresis worsen in their later life (16, 20).

Moreover, Enuresis children may develop anxiety disorders like panic attacks, mood disorders, severe depression (16, 20), school rejection, poor academic performance, and poor social relationships (21).

It is unclear what precise mechanism connects sleep-disordered breathing to the higher likelihood of NE. Potential independent risk factors for NE in school-age children include male gender, obesity, early bedtime, loud snoring, breathing difficulties, and mouth breathing at night (22).There are several contributing factors to the nocturnal enuresis, such as poor self-esteem, family stress, social isolation, family history, being male, increase family size, recurrent UTI, developmental delay who had down syndrome, socio-economic status, maternal educational status, age of mothers during birth, children exposed for stressful events were significant predictors of nocturnal enuresis which was found in different studies (21, 23–27).

A child's sleep diary provides fascinating insights into their self-perception, while actigraphy offers supplementary information regarding their sleep patterns at night. Actigraphy in combination with sleep diary could provide a valuable method of assessing factors affecting subjective and objective sleep assessments (28).

In addition, nocturnal enuresis causes social, psychological, and economic burdens on a country. Nocturnal enuresis such as schools can be anticipated and managed among school-aged children to yield healthy productive generations in the future. Therefore, identifying the current prevalence of nocturnal enuresis and its risk factors is important to develop targeted strategies and interventions. Moreover, to the best of the researchers, despite different studies conducted in several developed countries, there were limited studies of factors associated with nocturnal enuresis in Ethiopia, particularly in the present study area. Therefore, this study aimed to assess the prevalence of nocturnal enuresis among children aged 5–14 years in Gondar City, Northwest Ethiopia, and its predictors.

## Methods

### Study setting and design

A community-based, Cross-sectional study design was conducted from April 1, 2023 to April 30, 2023 in Gondar city. Gondar is located in the northwest of the Amhara region, approximately 741 kilometers from Addis Ababa, the capital city of Ethiopia. In Gondar city, there are six sub-cities: Maraki, Arada, Zobel, Azezo-teda, Fasil, and Jantekel. There are 36 Keble's (11 rural and 25 urban) found in Gondar city. It has one comprehensive specialized hospital, one General hospital, eight health centers, 14 health posts, and over 50 private clinics. According to the Gondar health offices report, in 2022, Gondar city will have 11,0505 households. The total population was 475,172. Among these 108, 033 of the population are children aged between 5 and 14 years. The study used all caregivers with children aged 5–14 years who live in Gondar city as the source population and all caregivers with all children aged 5–14 years who live in selected Keble and present during the data collection period as the study population. Children diagnosed with diabetes mellitus and not residing in Gondar city for less than 6 months were excluded from the study.

### Sample size determination

The sample size was calculated using, single population proportion formula by considering the following statistical assumptions.

*P* = proportion of Enuresis 8.6% which conducted in Bahrdar city (29). Z *α*/2 = Z score of 95% CI, d = Margin of error (3%).

$$\mathrm{Sample}\;\mathrm{size}=\frac{z^{2}p\left(1-p\right)}{w^{2}}=\frac{1.96^{2}0.086\left(1-0.086\right)}{0.03^{2}}=335$$

Where *n* = sample size =335

The critical value for normal distribution at a 95% confidence level which equals 1.96 *P* = Non response rate = 10% = 34.

The total sample size is *n* = 369.

The design effect was used and multiplied by two which, giving a total a sample size.

### Sampling technique and procedure

**Figure 1:**
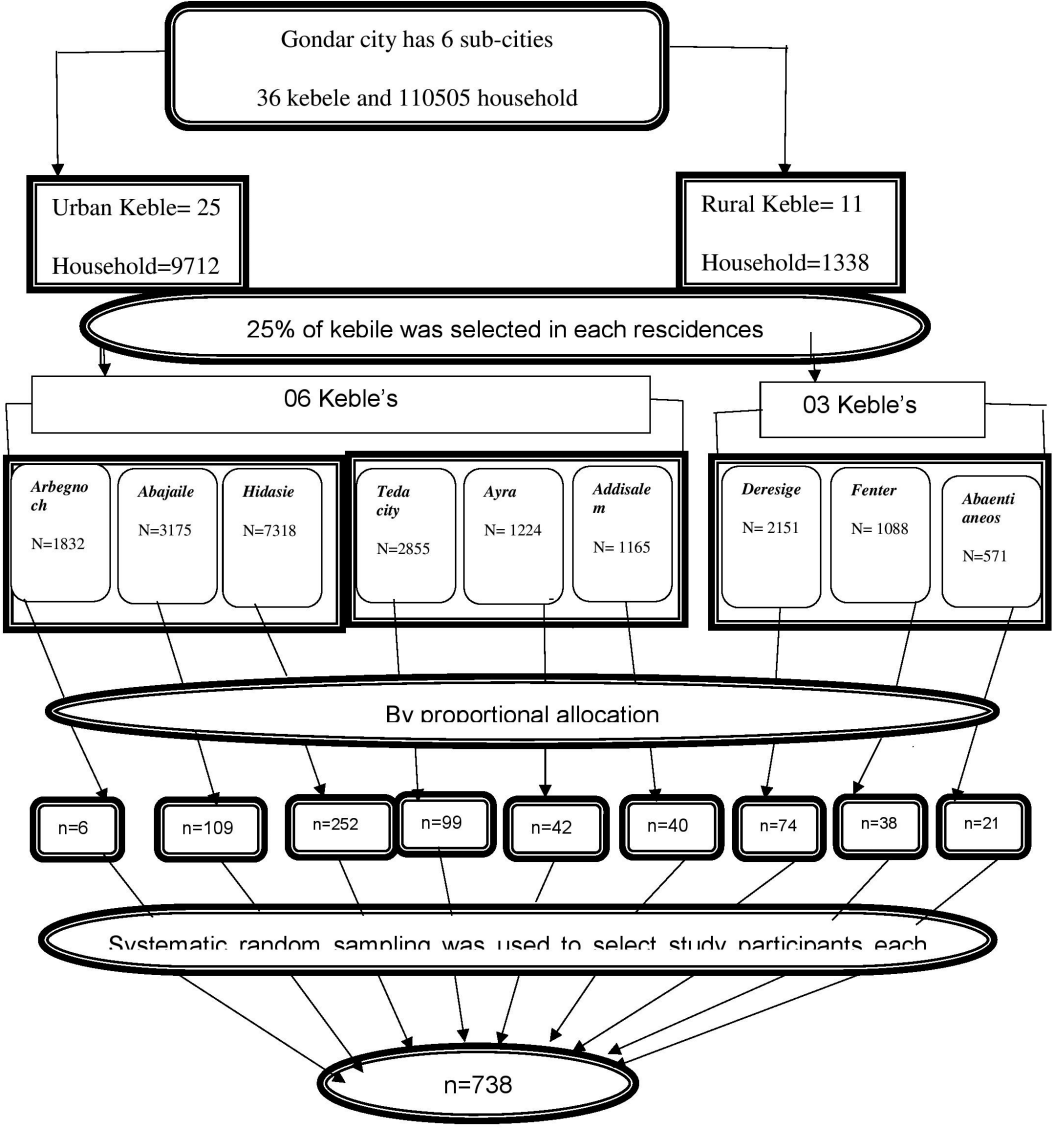
Schematic presentation of sampling procedure to assess prevalence and associated factors of nocturnal enuresis among children aged 5–14 years in Gondar City, Northwest Ethiopia, 2023.

In Gondar city, there are six sub-cities that have 36 Kebles's (25 urban and 11 rural Keble's). Among urban and rural Keble 25% of Keble were selected in each residency (6 Keble from urban and 3 Keble from rural). A proportional allocation technique was used to select the number of study subjects in each selected keble. After labeling households who had a child aged 5–14 years in each selected Keble's systematic random sampling technique, every K interval was used to select the actual sample. If there was more than one child aged 5–14 years in the selected household, the lottery method was applied to select study participants. Multistage stratified random sampling was used. K = *N*/*n* = 21,378/738 = (on lottery method 11 was selected as the starting point of the K interval (Figure 1).

### Operational definition

#### Nocturnal enuresis

Accordingto the DSMV (diagnostic and statistical manual of mental disorders V) criteria, nocturnal enuresis is defined as the urination of children over the age of five who do so twice each week for three consecutive months in clothing or in bed. It can happen at night, during the day, or a combination of both (30).

#### Child had nocturnal enuresis

The child had nocturnal enuresis when bed was wet during the night at least twice a week for three consecutive months, otherwise not (no).

Psychiatric disorders and mental disorders- diagnosed by psychiatrists according to the Diagnostic and Statistical Manual of Mental Disorders (DSM-5) (31).

Exposure to stressful events, including death of a loved one, parents by divorce, moving/leaving a permanent home, and presence of long-term illness (32).

School performances—assessed by considering such issues as not going to certain classes during the day, Trouble finishing home work, and assignments Low grades in one or more class Not wanting to talk about school or show a report card Saying he or she is bored in class or cannot keep up with the teacher (33).

Constipation: characterized by infrequent painful defecation, fecal incontinence, abdominal pain, and hard small stool usually less than three times per week and diagnosed by a physician (34).

Urinary tract infection (UTI)- is the presence of pyuria dysuria, frequency, urgency, suprapubic discomfort, and flank pain and is diagnosed by a physician (35).

Toilet training is the practice of teaching a child to recognize his or her body signals for urinating, having a bowel movement, and using a potty chair or toilet correctly and at the appropriate time (36).

Caffeine-containing drinks include coffee, cocoa, checkolets, and coffee containing foods (37). If the child takes one of the caffeine-containing drinks, we said yes otherwise not.

### Variables

The Dependent variable of this study was Nocturnal enuresis (Yes/No) and the independent variables were Children socio-demographic factors (Socio demographic variable includes age, sex, birth order, and number of siblings, family size, and residency), Parent socio-demographic factors (Parent socio-demographic variable includes mothers’ age, mother education status, mother occupational status, fathers’ age, father education status, father occupational status, parent’s marital status, family income), and family history and health-related factors (family history and health-related includes toilet training, drinking caffeine contains drink, family history of NE, exposure to a stressful event, presences of mental/psychiatry disease, sleep problem, number of people sleeping in one room, presences of constipation, presences urinary tract infection, habit of voiding before bed, poor school performances).

### Data collection tool and procedure

A structured and pretested interviewer-administered, Amharic version of the interviewer administered questionnaire was used to collect data from the study participants. The data was collected for 30 consecutive days. The questionnaire was adapted from previous comparable studies. The questionnaire has three sections. The first section discusses the sociodemographic characteristics of the children and parents adapted from previous studies (7, 9, 29). The second section includes family history and health-related characteristics adapted from previous studies (7, 9, 29),and the third section is a questionnaire to assess outcome variable(NE) adopted from previous comparable studies (7, 9, 29). Three diploma nurses were assigned for data collection; one BSc nurse was assigned to supervise of the entire data collection. The data collector was informed to collect data after obtaining informed consent from the parents of the study subjects.

### Data quality assurance

The questionnaire was prepared in English and then translated into Amharic and then back to English by a language expert to check its consistency and contextualization. Before the actual data collection, a pre-test was performed on 5% (36 samples) of the total study-appropriate participants at Bahirdar city Keble 07 who had the same characteristics. The pretest was conducted 2 weeks before the actual data collection period. The results of the pretest were discussed and all necessary amendments was made to the, instructions, contents, order, and grammatical issues based on the pretest results. Tool validity and reliability were maintained by using pre-validated in reliable tool used by previous studies and by using expertise opinion. All data were checked for completeness, accuracy, clarity, and consistency by the supervisors and investigators each night after the data were collected. Investigators and supervisors closely monitor the data collection process. Intensive training was given to data collectors and supervisors for one day by the principal investigator regarding the objectives, tools, and processes of data collection and how to maintain the confidentiality of the study subject. During the data collection period, the supervisors’ responsibility was to check whether the questionnaire was correctly filled or not and to make closely supervision of data collectors then the principal investigator then performed spot-checking and reviewed the data collection process completed daily to ensure completeness and consistency of the information that was collected. Furthermore, data was also checked during entry into the computer before analysis.

### Data processing and analysis

The data were coded, cleaned, edited, and entered Epi data version 4.6.02 to minimize logical errors and design skipping patterns. Then, the data were exported to SPSS window version 25 for analysis. Summary statistics (mean or median) for continuous variables and percentage and frequency for categorical variables were used to describe the study population regarding the relevant variable. Tables and figures were used to present the data. All variables with *P* ≤ 0.25 in the Bivariable analysis were included in the final model of multivariate analysis the multivariate logistic regression was used to assess the association between independent and the outcome variable. The model goodness of fit was tested by Hosmer-Lemeshow statistic tests and revealed *p*-value = 0.856 and multi-collinearity was checked by a variances inflation factor (VIF),which revealed 2.75. The backward method of variable selection method was used to run multivariable logistic regression. The direction and strength of statistical association was measured. Theadjusted odds ratio along with 95% CI was estimated, and a *P*-value <0.05 was considered statistically significant.

### Ethical consideration

Ethical clearance was obtained from the University of Gondar, College of medicine and health sciences research and community services offices and submitted to the Gondar city mayor, selected Keble leader to communicate with the purpose of study, duration of the study, and way of maintaining confidentiality. All study participants were informed about the purpose of the study and their right to refuse. Written and signed voluntary consent was obtained from the study participants prior to data collection. The respondents were also told that the information obtained from them was treated with complete confidentiality and did not cause any harm to them. Confidentiality of the study participants was maintained using codes, and no other body will see each finding. In addition, data collectors are health professionals who are announced for a way of maintaining confidentiality. After obtaining their permission, our actual data collection was conducted.

## Results

### Sociodemographic characteristics of the participants

A total of 730 study participants participated in the study, which makes a response rate of 98.92%. The age of children ranged from 5 to 14 years; the mean age was 8.35 years (standard deviation: ± 2.77 years). most study participants, 427 (58.5%%), were age range from 5 to 8 years. More than half of study participants 433 (59.3%) were girls. Regarding birth order of child, 278 (38.1%), 288 (39.5%), 117 (16%), and 47 (6.4%) had first, second, third, fourth, and above birth order, respectively. 440 (60.3%) of children live from urban residency (Table 1).

**Table 1:** Socio-demographic characteristics of children**’**s age 5–14 years in Gondar City, Northwest Ethiopia, 2023 (*n* = 730).

Variables	Category	Frequency	Percentage
Age of child	5–8 year	427	58.5
	9–12 year	220	30.1
	13–14 year	83	11.4
Sex	Female	433	59.3
	Male	297	40.7
Birth order	First	278	38.1
	Second	288	39.5
	Third	117	16.0
	Fourth and above	47	6.4
Residency	Rural	290	39.7
	Urban	440	60.3
Number of sibling	No sibling	85	11.6
	0	9	1.2
	1–2 siblings	155	21.2
	>=3 siblings	422	57.8

### Sociodemographic characteristics of the parents

The childes Mothers’ age ranges from 22 to 52 years, and the mean age of mother was 34.46-year (SD ± 6.63 year). The child fathers’ age ranges from 27 to 62 years, and the median age of fathers were 40.0 year (IQR = 9.0) year. The majority 422(57.8%) of mothers’ ages ranged from 30 to 39 years. Only 17.3% of Mothers reported that they were unable to read and write, while 21.2% of fathers ‘reported that they were unable to read and write. Of the 730 participants, 400 (54.8%) of Mothers’ and 191 (26.2%) of fathers were housewives and farmers, respectively. Of the 730 participants, 517 (70.8%) of the parents lived together (Table 2).

**Table 2:** Socio-demographic characteristics of parents of children age 5-14 year in gondar city, northwest Ethiopia, 2023 (*n* = 730).

Variables	Category	Frequency	Percentage
Mothers’ age	18–20 year	9	1.2
	21–29 year	155	21.2
	30–39 year	422	57.8
	>=40 year	144	19.7
Mothers’ educational status	Unable to read and write	126	17.3
	Primary school	202	27.7
	Secondary school	155	21.2
	Collage and above	247	33.8
Mothers’ occupational status	Housewife	400	54.8
	Daily laborer	45	6.2
	Governmental employee	161	22.0
	Non-governmental employee	14	1.9
	Merchant	74	10.1
	Other	36	4.9
Fathers’ age	18–24 year	18	2.4
	25–35 year	136	20.2
	36–45 year	384	52.6
	>=46 year	182	24.9
Fathers ‘educational status	Unable to read and write	155	21.2
	Primary school	128	17.5
	Secondary school	117	16.0
	Collage and above	330	45.2
Fathers’ occupational status	Farmer	191	26.2
	Daily laborer	81	11.1
	Governmental employee	226	30.9
	Non-governmental employee	32	4.4
	Merchant	137	18.8
	Other	63	8.6
Parents’ marital status	Live together	517	70.8
	Live separated/	83	11.4
	Divorced	92	12.6
	Windowed	38	5.2
Family monthly income	<2,000	137	18.8
	>=2,000 ETB	593	81.2

### Family history and health-related characteristics

**Figure 2:**
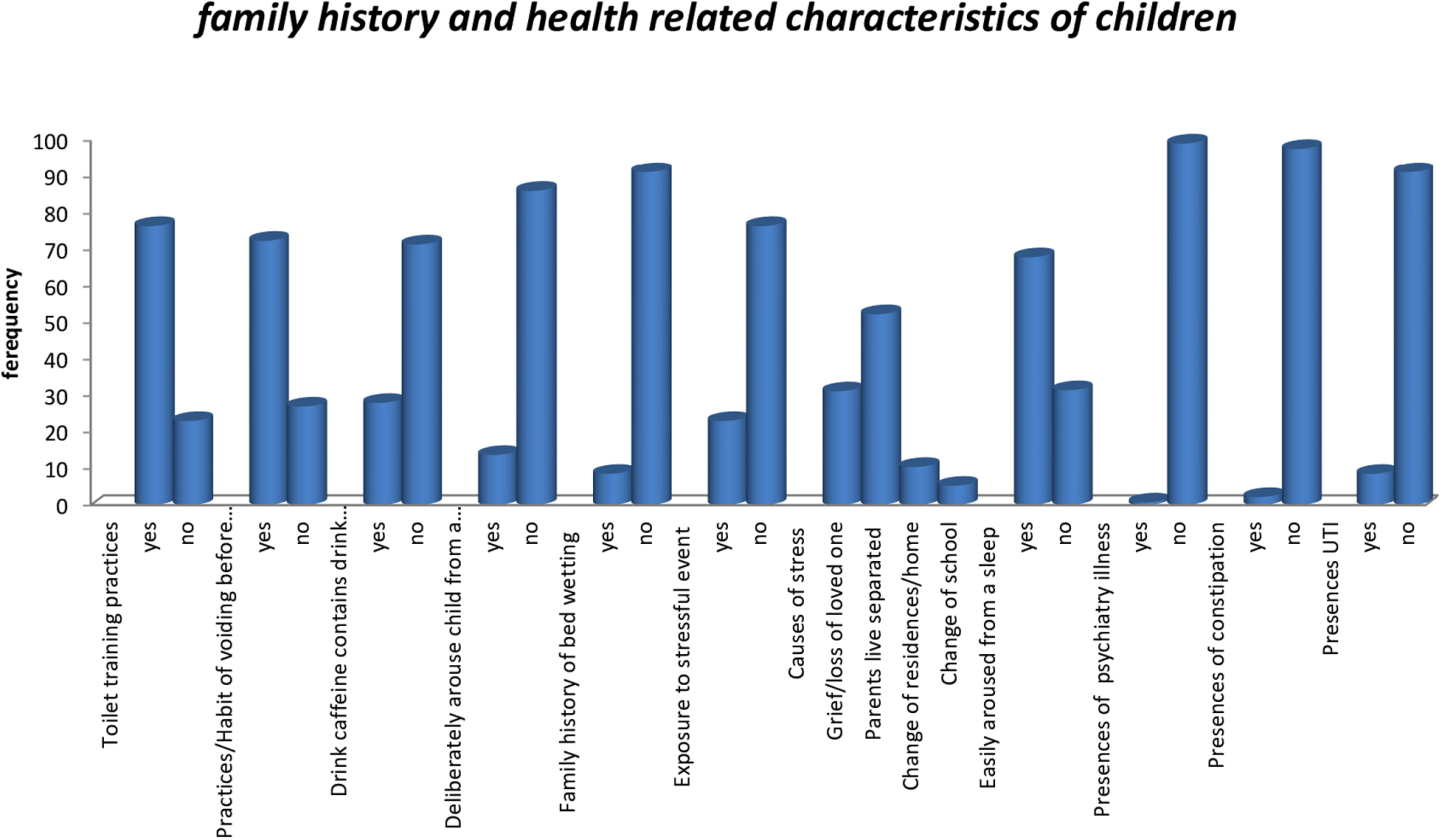
Family history and health related characteristics of children aged 5–14 years in Gondar City, northwest Ethiopia, 2023.

Most study participants (559 (76.6%) had toilet training practices, and 200 (27.4%) of children had practices voiding before bed. Of the 207 (28.4%) study participants who consumed caffeine before bed. 63 (8.6%) had a family history of nocturnal enuresis. The 171 (23.4%) study participants had a history of exposure to stressful events. The majority of (52.6%) stress occurs because parents live separated. In addition, 8.4% of children had urinary tract infection diagnosed by physician (Figure 2).

### Prevalence of nocturnal enuresis

**Figure 3:**
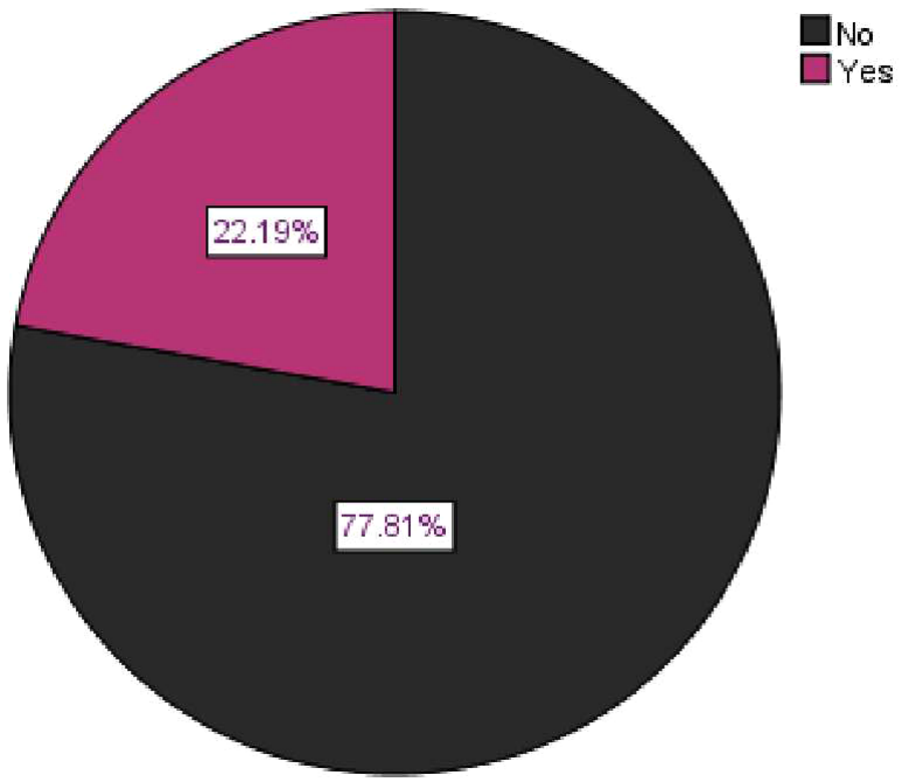
Overall prevalence of nocturnal enuresis among children aged 5–14 years in Gondar City, northwest Ethiopia, 2023.

The overall prevalence of nocturnal enuresis among children aged 5–14 years was 162(22.2%). (Figure 3)

### Factors associated with nocturnal enuresis

First, a bivariable analysis was performed to identify the determinants of nocturnal enuresis. Accordingly, sex, age of children, fathers’ age, residences, toilet training practices, habit of voiding before bed, drinking caffeine before bed, presence of stressful events, and school performance were eligible for the multi-variable analysis (*p* < 0.25). In the multivariable model, sex of child, Toilet training practices, drinking caffeine-containing drinks before bed, and presence of stressful events were significant predictors of nocturnal enuresis among children's age 5–14 years in Gondar city (*p* < 0.05).

The odds of having nocturnal enuresis among boys was 46% lower than that among girls [AOR = 0.54; 95% CI (0.31, 0.93)]. Regarding toilet training practices, children who had no toilet training practices had 2.5 times the risk of developing nocturnal enuresis than children who had toilet training practices [AOR = 2.50; 95% CI (1.02, 6.15)]. Children who had no caffeine-containing drink before bedtime had an 84% lower risk of developing nocturnal enuresis compared with children who had caffeine-containing drink before bedtime [AOR = 0.16; 95% CI (0.09, 0.29)]. Children who experienced exposure to stressful events had 20 times the risk of developing nocturnal enuresis compared with children who had no exposure to stressful events [AOR = 20; 95% CI (11.12, 33.34)]. (Table 3)

**Table 3:** Bivariable and multivariable logistic regression of factors associated with nocturnal enuresis among children age 5–14 years in Gondar City, 2023 (*n* = 730).

Variables	Category	Nocturnal enuresis		COR with 95% CI	AOR with 95% CI	*p*- value
		Yes	No			
Sex	Male	36	261	0.34 (0.23,0.50)*	0.54 (0.31,0.93)	**0** **.** **026** *
	Female	126	307	1	1	
Fathers age	18–24 year	5	15	1.11 (0.34,3.53)	0.61 (0.11,3.54)	0.577
	25–35 year	50	91	2.11 (1.27,3.49)*	1.45 (0.66, 3.19)	0.355
	36–45 year	72	325	0.85 (0.54,1.33)	1.34 (0.67,2.70)	0.411
	>=46 year	36	138	1	1	
Age of child	5–8 year	108	319	2.78 (1.35,5.75)*	1.11 (0.41,2.95)	0.848
	9–12 year	45	175	2.11 (0.98,4.55)	0.74 (0.25,2.19)	0.580
	13–14 year	9	74	1	1	
Residences	Rural	81	209	1.72 (1.21,2.44)	1.09 (0.63,1.87)	0.764
	Urban	81	359	1	1	
School performances	Poor	45	72	2.65 (1.74,4.05)	0.52 (0.22, 1.27)	0.153
	Good	117	496	1	1	
Toilet training practices	No	81	90	5.31 (3.63,7.78)	2.50 (1.02,6.15)	0.045*
	Yes	81	478	1	1	
Drinking caffeine contain drink	No	72	451	0.21 (0.14,0.30)	0.16 (0.09,0.29)	<0.001**
	Yes	90	117	1	1	
Presences of stressful event	Yes	108	63	16.03 (10.55,24.37)	20.11 (11.12,33.34)	<0.001**
	No	54	505	1	1	
Habit of voiding before bed	No	81	119	3.77 (2.61,5.45)	1.82 (0.87,3.79)	0.114
	Yes	81	449	1	1	

AOR, adjusted odds ratio; CI, confidence interval; COR, crude odds ratio.

* Statistically significant at *p*-value <0.05.

** Highly statistically significant <0.001.

## Discussion

To alleviate the problem of enuresis in children, a wide variety of management schemes are available. As part of the treatment process, various techniques can be used, such as behavioral therapy, such as waking a child to a void in the toilet, dry bed training, reward systems, acupuncture, bed alarm therapy, electrical stimulation, Tuina (traditional Chinese medicine), and desmopressin (38–44).

The purpose of this study was to assess the prevalence and associated factors of nocturnal enuresis among children 5–14 years in Gondar city, Ethiopia. The result of the current study showed that 162 (22.2%) (CI = 19.2%, 25.1%) children had nocturnal enuresis, which is in line with the study done in Addis Ababa, Ethiopia (20.8%) (8).

In this study, the prevalence of nocturnal enuresis was higher than the study done in Iran, (10.2%) (45), China (3.9%) (4), Khorramabad (8%) (46), Rafsanjani (10.6%) (47), Turkey (17.5%) (5), and Bahrdar city, Ethiopia (8.6%) (29). A possible explanation for this discrepancy might be differences in sample size, study setting and geographical and population differences. The study in Bahrdar, Ethiopia, was conducted only on children who had primary nocturnal enuresis, this study had no reported-on children who had secondary nocturnal enuresis and this may be the reason for the discrepancy discrepancy.

In this study, the prevalence of nocturnal enuresis was lower than that reported in Yemen (28.6%) (48), Saudi (31.2%) (49) and Adama city, Ethiopia (26.6%) (9). A possible justification for this discrepancy might be differences in sample size, and study area, differences in study participants. The study in Yemen and Adama city was conducted among children age 6–15 years while in this study the study subject was children age 5–14 year, this may be the reason for the discrepancy.

The current study showed that sex, toilet training practices, drinking caffeine before bed, school performances, and exposure to stressful events were the main factors associated with nocturnal enuresis among children age 5–14 years in Gondar city, Ethiopia.

Children age 5–14 year who had taken caffeine before bed had high risk of developing nocturnal enuresis than thus had not taken before bed which supported by the study done in Egypt (7). Studies have shown that caffeine is a mild diuretic, which results in the kidneys releasing more water from the bloodstream, as a result the bladder becomes filled and urination can occure (50). In addition, caffeine-containing drinks cause irritation the bladder, so urination may occur (50).

In this finding, Children exposed to stressful events had a higher risk of developing nocturnal enuresis than those who had no exposure to stressful events, which is supported by the study done in Yemen (48). Emotional stress brought on by unpleasant experiences or disruptions in your daily routine can cause bedwetting (51). Episodes of bedwetting can be triggered by moving from a permanent home, starting school, losing a loved one, or experiencing sexual abuse (51). Children who experience stress have a more challenging time falling asleep, leading to sleep deprivation. After being sleep deprived, they go into an even deeper rest where they cannot wake themselves up to use the bathroom and do not urinate before bedtime (52, 53).

Girls had a higher risk of developing nocturnal enuresis than boys, which supported by the study in Thailand (54). However, this finding contradict the study done in Addis Ababa (8). A possible reason for this discrepancy might be differences in sample size, study setting, study population, and inclusion and exclusion criteria.

In this study, children who had no toilet training practices seven times risk of developing nocturnal enuresis than had toilet training practices which is consistencies with the study in Kenya (55) and Turkey (56). A possible explanation could be that children who began using the toilet at the age of 24months benefit from healthy bladder function and urine storage, as well as from toilet training practices that encourage urination before bed and sound an alarm when the bladder is full with urine (51).

In this study, age of child residences, parental marital status, mothers and fathers’ occupational and educational status, family history of nocturnal enuresis, presences of UTI, presences of constipation and presences of psychiatry ill ness had not a significant association with child nocturnal enuresis. The possible reason for this discrepancy may be differences in the method, inclusion and exclusion criteria, and differences in study participants and study stetting.

### Strengths and limitations of the study

This study has a large sample size, which can provide more accurate prevalence estimates. A study with a sufficiently large sample size can increase the representativeness of the findings and enhance their generalizability.

The issue of NE is sensitive and potentially embarrassing for children. Reporting by parents may introduce bias because parents may underreport or over report.

The perception and reporting of nocturnal enuresis can be affected by cultural and social factors. People's attitudes toward discussing bedwetting openly, cultural norms, and social stigma can vary across different populations, potentially affecting the prevalence of bedwetting.

Since depression was not considered as a contributing component in this study, future researchers on the subject can include it and demonstrate the reciprocal relationship between them.

## Conclusion

In this study, the prevalence of nocturnal enuresis children age 5–14 years was higher than that in previous studies. Sex of child, Toilet training practices, drinking caffeine-containing drinks before bed, and presence of stressful events were significant predictors of nocturnal enuresis. In light of this, initiatives should be considered to reduce the incidence of nocturnal enuresis.

## Recommendation

### To the federal minister of health of Ethiopia

The federal health minister of Ethiopia should engage with stockholders working on children's health to reduce the frequency of nocturnal enuresis among children. Emphasis should be placed on health education and promotion concerning nocturnal enuresis by using media and brochures on toilet training practices, ways of coping with stressful events, and reducing forceful punishment for their child.

### To Gondar city administrations health offices

The study city administration should engage with stockholders working on children's health to reduce the frequency of nocturnal enuresis among children. Emphasis is placed on health education and promotion concerning nocturnal enuresis by using media brochures and health extension workers on toilet training practices and ways of coping with stressful events. Furthermore, school-based health education for parents and their children on nocturnal enuresis is required to reduce the prevalence and consequences of nocturnal enuresis.

For researcher: The authors recommended performing research on Nocturnal enuresis qualitatively.

## Data Availability

The original contributions presented in the study are included in the article/Supplementary Material, further inquiries can be directed to the corresponding author.

## References

[1] Nijman R Bower W Butler U Ellsworth P Tegkul S Von Gontard A. Diagnosis and management of urinary incontinence and encopresis in childhood. Incontinence. (2005) 2:965–1058.

[2] Nevéus T von Gontard A Hoebeke P Hjälmås K Bauer S Bower W The standardization of terminology of lower urinary tract function in children and adolescents: report from the standardisation committee of the international children’s continence society. J Urol. (2006) 176(1):314–24. 10.1016/S0022-5347(06)00305-3 16753432

[3] Hunskaar S Burgio K Clark A Lapitan M Nelson R Sillen U Epidemiology of urinary (UI) and faecal (FI) incontinence and pelvic organ prolapse (POP). Incontinence. (2005) 1:255–312.

[4] Huang H-M Wei J Sharma S Bao Y Li F Song J-W Prevalence and risk factors of nocturnal enuresis among children ages 5–12 years in Xi’an, China: a cross-sectional study. BMC Pediatr. (2020) 20:1–8. 10.1186/s12887-020-1908-6 32571248 PMC7310244

[5] Ozden C Ozdal OL Altinova S Oguzulgen I Urgancioglu G Memis A. Prevalence and associated factors of enuresis in turkish children. Int Braz J Urol. (2007) 33:216–22. 10.1590/S1677-55382007000200013 17488542

[6] Fockema MW Candy GP Kruger D Haffejee M. Enuresis in South African children: prevalence, associated factors and parental perception of treatment. BJU Int. (2012) 110(11c):E1114–E20. 10.1111/j.1464-410X.2012.11416.x 22958477

[7] Hamed A Yousf F Hussein MM. Prevalence of nocturnal enuresis and related risk factors in school-age children in Egypt: an epidemiological study. World J Urol. (2017) 35:459–65. 10.1007/s00345-016-1879-2 27306687

[8] Desta M Hägglöf B Kebede D Alem A. Socio-demographic and psychopathologic correlates of enuresis in urban Ethiopian children. Acta Paediatr. (2007) 96(4):556–60. 10.1111/j.1651-2227.2007.00229.x 17306004

[9] Ibrahim N Tolessa D Mannekhulihe E. Prevalence and factors associated with enuresis among children in adama city, oromia regional state. Ethiopia. Int J Physiatry. (2021) 7:021. 10.23937/2572-4215.1510021

[10] Djurhuus JC Rittig S. Nocturnal enuresis. Curr Opin Urol. (2002) 12(4):317–20. 10.1097/00042307-200207000-00010 12072653

[11] Butler RJ. Night wetting in children: psychological aspects. J Child Psychol Psychiat Allied Discip. (1998) 39(4):453–63. 10.1017/S0021963098002224 9599774

[12] Butler RJ. Nocturnal enuresis: the child’s experience. Elsevier - Health Sciences Division. (1994):179–97.

[13] Hägglöf B Andren O Bergström E Marklund L Wendelius M. Self-esteem in children with nocturnal enuresis and urinary incontinence: improvement of self-esteem after treatment. Eur Urol. (1998) 33(Suppl. 3):16–9. 10.1159/000052236 9599731

[14] Moffatt MEK Kato C Pless IB. Improvements in self-concept after treatment of nocturnal enuresis: randomized controlled trial. J Pediatr. (1987) 110(4):647–52. 10.1016/S0022-3476(87)80572-3 3550026

[15] Gong S Khosla L Gong F Kasarla N Everaert K Weiss J Transition from childhood nocturnal enuresis to adult nocturia: a systematic review and meta-analysis. Res Rep Urol. (2021) 13:823–32. 10.2147/RRU.S302843 34858887 PMC8631987

[16] Tsuji S Takewa R Ohnuma C Kimata T Yamanouchi S Kaneko K. Nocturnal enuresis and poor sleep quality. Pediatr Int. (2018) 60(11):1020–3. 10.1111/ped.13703 30257061

[17] Caldwell PH Deshpande AV Von Gontard A. Management of nocturnal enuresis. Br Med J. (2013) 347. 10.1136/bmj.f6259 24170156

[18] Robson WLM. Current management of nocturnal enuresis. Curr Opin Urol. (2008) 18(4):425–30. 10.1097/MOU.0b013e3282fcea9c 18520767

[19] Redsell S Collier J. Bedwetting, behaviour and self-esteem: a review of the literature. Child Care Health Dev. (2001) 27(2):149–62. 10.1046/j.1365-2214.2001.00195.x 11251613

[20] Kuwertz-Bröking E von Gontard A. Clinical management of nocturnal enuresis. Pediatr Nephrol. (2018) 33(7):1145–54. 10.1007/s00467-017-3778-1 28828529

[21] Touchette É Petit D Paquet J Tremblay RE Boivin M Montplaisir JY. Bed-wetting and its association with developmental milestones in early childhood. Arch Pediatr Adolesc Med. (2005) 159(12):1129–34. 10.1001/archpedi.159.12.1129 16330736

[22] Alshehri AA Zaki MSH Nour SO Gadi WH Zogel BA Alfaifi SM Sleep-disordered breathing and its association with nocturnal enuresis at the primary schools in Saudi Arabia: a cross-sectional study. Children. (2023) 10(6):1074. 10.3390/children10061074 37371305 PMC10296854

[23] Longstaffe S Moffatt ME Whalen JC. Behavioral and self-concept changes after six months of enuresis treatment: a randomized, controlled trial. Pediatrics. (2000) 105(Supplement_3):935–40. 10.1542/peds.105.S3.935 10742350

[24] Wang QW Wen JG Zhu QH Zhang GX Yang K Wang Y The effect of familial aggregation on the children with primary nocturnal enuresis. Neurourol Urodyn. (2009) 28(5):423–6. 10.1002/nau.20666 19012298

[25] Rona RJ Li L Chinn S. Determinants of nocturnal enuresis in England and Scotland in the'90s. Dev Med Child Neurol. (1997) 39(10):677–81. 10.1111/j.1469-8749.1997.tb07362.x 9352729

[26] Yeung C Sit F To L Chiu H Sihoe J Lee E Reduction in nocturnal functional bladder capacity is a common factor in the pathogenesis of refractory nocturnal enuresis. BJU Int. (2002) 90(3):302–7. 10.1046/j.1464-410X.2002.02884.x 12133069

[27] Lawless MR McElderry DH. Nocturnal enuresis: current concepts. Pediatr Rev. (2001) 22(12):399–407. 10.1542/pir.22-12-399 11731679

[28] Mazza S Bastuji H Rey AE. Objective and subjective assessments of sleep in children: comparison of actigraphy, sleep diary completed by children and parents’ estimation. Front Psychiatry. (2020) 11:495. 10.3389/fpsyt.2020.00495 32587532 PMC7297917

[29] Amare T Zewde F. Associated factors of primary enuresis among children and adolescents in Amhara Region, Northwest, Ethiopia, 2016. J Pediatr Nephrol. 2018;6(1):1–8.

[30] von Gontard A. The impact of DSM-5 and guidelines for assessment and treatment of elimination disorders. Eur Child Adolesc Psychiatry. (2013) 22(Suppl 1):61–7. 10.1007/s00787-012-0363-9 23247389

[31] Guha M. Diagnostic and statistical manual of mental disorders: dSM-5. Reference Reviews. (2014):36–7. 10.1108/RR-10-20130256

[32] Reiss F Meyrose A-K Otto C Lampert T Klasen F Ravens-Sieberer U. Socioeconomic status, stressful life situations and mental health problems in children and adolescents: results of the German BELLA cohort-study. PLoS One. (2019) 14(3):e0213700. 10.1371/journal.pone.0213700 30865713 PMC6415852

[33] Karande S Kulkarni M. Poor school performance. Indian J Pediatr. (2005) 72:961–7. 10.1007/BF02731673 16391452

[34] Jin L Deng L Wu W Wang Z Shao W Liu J. Systematic review and meta-analysis of the effect of probiotic supplementation on functional constipation in children. Medicine (Baltimore). (2018) 97(39). 10.1097/MD.0000000000012174 PMC6181519 30278490

[35] Zorc JJ Kiddoo DA Shaw KN. Diagnosis and management of pediatric urinary tract infections. Clin Microbiol Rev. (2005) 18(2):417–22. 10.1128/CMR.18.2.417-422.2005 15831830 PMC1082801

[36] Choby BA George S. Toilet training. Am Fam Physician. (2008) 78(9):1059–64. 19007052

[37] Radwan A Al Jazairi A Qaddourah N Ahmed S Albrahim S Elhusein B Caffeine, Mental Well-Being, and Psychiatric Disorders. Nutrition and Psychiatric Disorders. Netherland: Springer (2022). p. 201–19.

[38] UK NCGC. Nocturnal enuresis: The management of bedwetting in children and young people. 2010.

[39] Neveus T Eggert P Evans J Macedo A Rittig S Tekgül S Evaluation of and treatment for monosymptomatic enuresis: a standardization document from the international children’s continence society. J Urol. (2010) 183(2):441–7. 10.1016/j.juro.2009.10.043 20006865

[40] Harris J Lipson A Dos Santos J. Evaluation and management of enuresis in the general paediatric setting. Paediatr Child Health. (2023) 28(6):362–8. 10.1093/pch/pxad023 PMC10517245 37744753

[41] Glazener CM Evans JH. Simple behavioural and physical interventions for nocturnal enuresis in children. Cochrane Database Syst Rev. (2004) 2. 10.1002/14651858.CD003637.pub2 15106210

[42] Azrin N Thienes P. Rapid elimination of enuresis by intensive learning without a conditioning apparatus. Behav Ther. (1978) 9(3):342–54. 10.1016/S0005-7894(78)80077-X

[43] Zheng-Tao L Song W Wu J Yang J Wang T Wu C-H Efficacy of acupuncture in children with nocturnal enuresis: a systematic review and meta-analysis of randomized controlled trials. Evidence-Based Complementary Altern Med. (2015) 2015. 10.1155/2015/320701 PMC4488007 26167190

[44] Tong C He Q Ho M Zhong Z Wu Q Chen M. Tuina for enuresis in children: a systematic review and meta-analysis of randomized controlled trials. Front Public Health. (2022) 10:821781. 10.3389/fpubh.2022.821781 35493365 PMC9039245

[45] Mohammadi M Raiegani AAV Jalali R Ghobadi A Salari N. The prevalence of nocturnal enuresis among Iranian children: a systematic review and meta-analysis. Urol J. (2019) 16(5):427–32. 10.22037/uj.v0i0.5194 31422577

[46] Bakhtiar K Pournia Y Ebrahimzadeh F Farhadi A Shafizadeh F Hosseinabadi R. Prevalence of nocturnal enuresis and its associated factors in primary school and preschool children of Khorramabad in 2013. Int J Pediatr. (2014) 2014. 10.1155/2014/120686 25374608 PMC4211300

[47] Torkashvand F Rezaeian M Bagheani T Zarafshan H Mostafavi S-A Bidaki R. Prevalence of nocturnal enuresis in school-age children in rafsanjan. J Pediatr Nephrol. (2015) 3(2):71–4. 10.22037/jpn.v3i2.7968

[48] Aljefri HM Basurreh OA Yunus F Bawazir AA. Nocturnal enuresis among primary school children. Saudi J Kidney Dis Transpl. (2013) 24(6):1233. 10.4103/1319-2442.121312 24231492

[49] Alhifthy EH Habib L Al-Makarem AA AlGhamdi M Alsultan D Aldhamer F Prevalence of nocturnal enuresis among Saudi children population. Cureus. (2020) 12(1). 10.7759/cureus.6662 31966951 PMC6964794

[50] Warzak WJ Evans S Floress MT Gross AC Stoolman S. Caffeine consumption in young children. J Pediatr. (2011) 158(3):508–9. 10.1016/j.jpeds.2010.11.022 21167501

[51] Woodward S. Bladder and bowel health. Redfern’s Nursing Older People. (2023) 315.

[52] Joinson C Sullivan S von Gontard A Heron J. Early childhood psychological factors and risk for bedwetting at school age in a UK cohort. Eur Child Adolesc Psychiatry. (2016) 25:519–28. 10.1007/s00787-015-0756-7 26294078 PMC4854940

[53] Esposito M Gallai B Parisi L Roccella M Marotta R Lavano SM Primary nocturnal enuresis as a risk factor for sleep disorders: an observational questionnaire-based multicenter study. Neuropsychiatr Dis Treat. (2013):437–43. 10.2147/NDT.S43673 23579788 PMC3621720

[54] Hansakunachai T Ruangdaraganon N Udomsubpayakul U Sombuntham T Kotchabhakdi N. Epidemiology of enuresis among school-age children in Thailand. J Dev Behav Pediatr. (2005) 26(5):356–60. 10.1097/00004703-200510000-00003 16222175

[55] Nzamu IM. Prevalence of Nocturnal Enuresis and Associated Factors among 6-14 Year old Children Attending Schools in a Rural District in Kenya. Kenya: University of Nairobi (2012).

[56] Gürnüš B Vurgun N Lekili M Işcan A Müezzinoǧlu T Büyüksu C. Prevalence of nocturnal enuresis and accompanying factors in children aged 7–11 years in Turkey. Acta Paediatr. (1999) 88(12):1369–72. 10.1111/j.1651-2227.1999.tb01146.x 10626524

